# Biopolymeric Capsules Containing Different Oils as Rejuvenating Agents for Asphalt Self-Healing: A Novel Multivariate Approach

**DOI:** 10.3390/polym14245418

**Published:** 2022-12-11

**Authors:** Jose L. Concha, Luis E. Arteaga-Pérez, Irene Gonzalez-Torre, Quantao Liu, Jose Norambuena-Contreras

**Affiliations:** 1 LabMAT, Department of Civil and Environmental Engineering, University of Bío-Bío, Concepción 4051381, Chile; 2 LPTC, Department of Wood Engineering, University of Bío-Bío, Concepción 4051381, Chile; 3 State Key Laboratory of Silicate Materials for Architectures, Wuhan University of Technology, Wuhan 430070, China

**Keywords:** self-healing, rejuvenators, alginate, encapsulation, factorial design, post hoc analyses

## Abstract

This study evaluated the effect of two encapsulation methods (i.e., dropping funnel and syringe pump), two concentrations of the alginate-based encapsulating material (2%, and 3%), and three oils as bitumen rejuvenators (virgin sunflower oil, waste cooking oil, and virgin engine oil) on the morphological, physical, chemical, thermal, and mechanical properties of encapsulated rejuvenators for asphalt self-healing purposes. A general factorial design 2 × 2 × 3 was proposed to design 12 different Ca-alginate capsules. Significant differences on the morphological, physical, and mechanical properties of the capsules were analysed by three-way ANOVA and Tukey HSD Post Hoc analyses. The effect of the type of oil on the self-healing capacity of cracked bitumen samples was also evaluated. The main results showed that the design parameters and their interactions significantly affected the morphological, physical, and mechanical properties of the capsules. Capsules synthesised via syringe pump method, with virgin cooking oil and 2% alginate was the most appropriate for asphalt self-healing purposes since its uniform morphology, encapsulation efficiency up to 80%, thermal degradation below 5% wt., and compressive strength above the reference asphalt compaction load of 10 N. Finally, the healing tests showed that virgin cooking oil can be potentially used as a rejuvenator to promote asphalt crack-healing.

## 1. Introduction

Asphalt pavements are commonly used for the construction of roads around the world. When exposed to rest periods, asphalt pavement has the potential to restore its stiffness and strength by closing the micro-cracks that occur when the pavement is subjected to traffic loads [1]. This property is known as self-healing ability, and it is conferred by the bitumen fraction in the asphalt mixture (about 5%wt.) [2,3,4]. Nonetheless, the natural self-healing in bitumen is a slow process due to low ambient temperature and asphalt ageing, while the ageing of asphalt is catalysed by several factors during the service life of the asphalt pavement [5,6]. Consequently, the mechanical properties of the asphalt pavement are affected, becoming more prone to temperature cracking and fatigue cracking, shortening the durability of pavement [7].

Traditionally, cracking of asphalt pavement caused by asphalt ageing has been treated using petroleum-based rejuvenators, via superficial application [8]. They consist of softening additives with a high proportion of organic components that aid in restoring the asphaltene/maltene ratio and the thermomechanical and rheological properties of the aged bitumen [9]. Nonetheless, the superficial application of rejuvenators on a pavement layer of 40–80 mm thickness [10] penetrated only 5 to 10 mm of a dense surface layer and no more than 20 mm of a porous surface layer [11]. To overcome this inefficiency, the incorporation of encapsulated bitumen rejuvenators into asphalt pavement have been developed over the past years, turning this topic into an emerging field of study [12]. The healing mechanism of the encapsulated rejuvenators is based on the rejuvenation of the aged binder [13]. The process is as follows: once a microcrack appears, the encapsulating material is broken by the fracture energy at the tip of the crack [14], resulting in the activation of the capsule; then, the bitumen rejuvenator is released, diffusing and permeating into the aged asphalt binder along the cracked surface; finally, the bitumen is softened, accelerating the healing process and reconstituting the aged asphalt binder’s chemical composition [15].

Different morphologies of encapsulated rejuvenators such as microcapsules [16], hollow fibres [17], saturated porous aggregates [18], and more recently, biobased spore microcapsules [19] have been developed for asphalt self-healing. In particular, spherical microcapsules with polynuclear internal structure have been widely studied, where different encapsulation methods, encapsulating materials, and bitumen rejuvenators have been proposed [12]. In reference to the encapsulation methods, dropping funnel [20] and syringe pressure pump [21] devices have commonly been used. In these methods, an oil-in-water emulsion (O/W), consisting of a disperse (oil droplets) and a continuous (encapsulating solution) phase, is extruded and precipitated in the form of droplets into a hardening solution (typically calcium chloride), resulting in the formation of capsules via ionic gelation principle. Among the encapsulating materials, sodium alginate biopolymer has lately become the most-used material because of its nontoxic, stable, and biodegradable properties [22]. Additionally, bitumen rejuvenators coming from (i) natural sources, e.g., sunflower [23] and rapeseed oil [24]; (ii) the automotive industry, e.g., virgin engine oil [25]; and the valorisation of waste from (iii) industrial sources such as pyrolytic oil from end-of-life tyres [26] and waste engine oil [20]; and (iv) natural sources such as waste vegetable oils [27] and bio-oils [21] have been successfully encapsulated for asphalt crack self-healing purposes.

The effectiveness of different encapsulated rejuvenators as bitumen healing agents has been proven in different bituminous materials with some promising results. Sun et al. [28] synthesised microcapsules based on an in situ polymerisation principle, using a light-oil as rejuvenator and melamine–urea–formaldehyde as the encapsulating agent. They concluded that bitumen with 5% wt. of microcapsules reached a strength–recovery ratio of up to 85%, enhancing the healing efficiency of asphalt binder. Xu et al. [29] synthesised capsules based on ionic gelation principle, using a commercial rejuvenator and polyethylene-alt-maleic–anhydride as the encapsulating agent. They showed that asphalt mortars with 2.6% wt. of capsules reached healing index up to 40%, indicating that the encapsulated asphalt rejuvenator can be released upon cracking and rejuvenate the aged material. Norambuena-Contreras et al. [30] synthesised capsules based on the ionic gelation principle, using sunflower oil as rejuvenator and Ca–alginate as the encapsulating agent. They showed that asphalt mixtures with 0.5% wt. of capsules reached a healing level up to 53%, being an effective additive for rejuvenating the aged matrix of asphalt materials.

Most encapsulated rejuvenators are synthesised under the one-factor-at-a-time approach. A drawback of this approach is that it does not consider the interactions that could exist between the capsules’ design parameters. Such interactions can simultaneously affect the physical and mechanical properties of the capsules to ensure their integrity when incorporated into bituminous materials. Thus, this approach can hardly respond questions such as (1) “Which is the appropriate encapsulation method for the synthesis of homogeneous-sized capsules for healing purposes?”; and (2) “What are the type and concentrations of the capsule components that optimise their physical, thermal, and mechanical performance?” This study proposes using a factorial approach to respond to these questions, providing a better understanding of how the main design parameters and their interactions affect the synthesis and the properties of the capsules. The main objective of this study is to evaluate the main and combined effect of the encapsulation method, alginate biopolymer concentration, and oil type on the morphological, physical, thermal, and mechanical properties of encapsulated rejuvenators for asphalt self-healing purposes. A general factorial design was performed considering two encapsulation methods, two alginate concentrations, and three types of oils to synthesise encapsulated rejuvenators at laboratory scale.

## 2. Materials and Methods

### 2.1. Materials

Polynuclear calcium–alginate capsules containing different oils were prepared in this study. Low-viscosity sodium alginate powder (Mannuronic/Guluronic ratio of 0.92, density 1.02 g/cm^3^, viscosity ≤ 300 mPa∙s in a 2% *w*/*w* solution), provided by Buchi (Flawil, Switzerland) and calcium–chloride dihydrate (CaCl_2_·2H_2_O) at 77% purity, provided by Winkler (Concepción, Chile) were used to form the polymeric matrix of the capsules. On the other hand, virgin cooking oil (VCO) from commercial sunflower oil; waste cooking oil (WCO) from recycled sunflower oil after one-cycle frying at 180 °C; and a commercial synthetic virgin engine oil (VEO)—code SAE 10W-40—were encapsulated as bitumen rejuvenating agents. The main properties of each oil are shown in Table 1. 

### 2.2. FTIR-ATR Test of the Encapsulated Oils

Rejuvenating oils, i.e., VCO, WCO and VEO, were chemically analysed by attenuated total reflection Fourier transform infrared spectroscopy (ATR-FTIR). The analyses were performed in a single-reflection Golden Gate^TM^ accessory (Specac, Orpington, UK) equipped with a diamond crystal. The IR spectra of oils were recorded over 32 scans in the range of 4000 cm^−1^ to 600 cm^−1^, with a resolution of 4 cm^−1^ using a Nicolet iS20 Spectrometer equipped with a DTGS detector and CaF_2_ windows (Thermo Fisher Scientific, Waltham, MA, USA). The comparison and analysis of spectra were performed after signal processing, i.e., baseline, ATR correction for Diamond crystal, peak identification, and normalisation ($a_{norm}$) according to the procedure presented by Hofko et al. [31]. Second-derivative spectra (Savitzky–Golay algorithm) were calculated to resolve the overlapping peaks and remove of the effects of baseline drifts.

### 2.3. Synthesis and Characterisation of the O/W Emulsions

Synthesis of the oil-based emulsions consisted of the following two steps: (S1) Based on the previous research literature on encapsulated rejuvenators [12], two types of sodium alginate solutions in concentrations of 2% *w*/*w* (viscosity of 205.0 cP @ 20 °C) and 3% *w*/*w* (viscosity of 602.1 cP @ 20 °C) of deionised water were prepared under continuous agitation using a mechanical stirrer (Scilogex, Model OS40-Pro-LB Pro, Rocky Hill, CO, USA) at 700 rpm for 30 min at room temperature. (S2) After this, VCO, WCO, and VEO were incorporated dropwise into each solution at a fixed biopolymer: oil mass ratio of 1:5, while stirring at 700 rpm for 40 min under mechanical agitation. In total, six O/W emulsions were prepared and labelled as X-Y, with X being the type of oil (VCO, WCO, and VEO) and Y the alginate concentration, i.e., 2% or 3%. Thus, an emulsion synthesised with VEO and 2% alginate concentration was labelled as VEO-2.

The viscosity of each emulsion was measured using a rotational viscometer (Fungilab, Model Smart Series, Barcelona, Spain). The physical stability of the emulsions (i.e., the tendency of the emulsion component to be physically separated over time), was characterised by the creaming index (CI), based on the method proposed by McClements [32] and recently applied by Norambuena-Contreras et al. [27]. CI was determined at: 0 h, 1 h, 2 h, 3 h, 6 h, 12 h, 24 h, 26 h, 28 h, and 30 h. Finally, representative values of viscosity and CI at each time were determined as the average of three measurements.

### 2.4. Synthesis and Characterisation of the Alginate-Based Capsules

**Figure 1:**
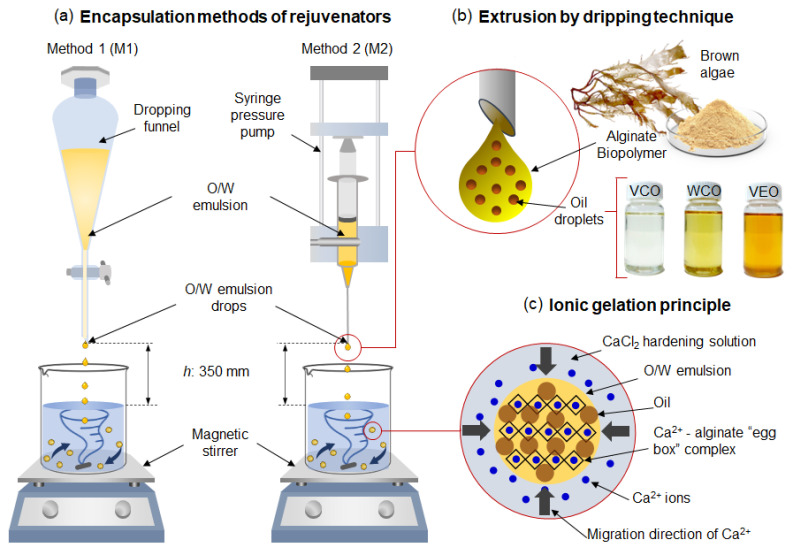
Scheme of the synthesis of the capsules under (**a**) dropping funnel and syringe pump methods. (**b**) Extrusion and releasing of an oil-in-water emulsion droplet. (**c**) Ca^2+^ migration and reaction with the alginate biopolymer by ionic gelation principle, forming the well-known Ca–alginate egg-box complex. As a result, the oil is encapsulated in the biopolymeric internal structure.

The synthesis of polynuclear capsules was based on the ionic gelation principle of sodium alginate in the presence of calcium ions. For that, two encapsulation methods were compared: the dropping funnel (M1) and the microfluidic pressure pump (M2), based on the parameters established by Norambuena-Contreras et al. [21], see Figure 1a.

First, a hardening solution consisting of 5% *w*/*w* of calcium chloride (CaCl_2_) into deionised water was prepared. Then, each emulsion design was poured into different recipients depending on the method: a 500 mL glass separatory funnel with internal diameter of 5.85 mm for M1, and a 60 mL syringe connected to a hollow steel needle of internal diameter of 1.2 mm for M2. For M1 the dropping mechanism depended on gravity and the viscosity of each emulsion, while for M2, the dropping mechanism depended on the flow rate (2 mL/min) provided by an automatic syringe pump (New Era NE-1010, Farmingdale, NY, USA), see Figure 1b.

For both methods, the emulsions were dropped into the CaCl_2_ hardening bath at 20 °C in constant agitation at 250 rpm using a magnetic stirrer. At this stage, the Ca^2+^ ions presenting in the calcium chloride solution reacted with the alginate-based emulsion by ionic gelation principle forming the well-known Ca–alginate egg-box complex, see Figure 1c, resulting in the encapsulation of the oil. Once the encapsulation process was finished, the capsules were maintained in the hardening bath for 30 min. Then, the capsules were filtered, rinsed with 300 mL of deionised water, and oven-dried for 24 h at 30 °C.

Finally, the capsules were placed into a glass vial and stored in a freezer at −18°C, preventing the oxidation of the oils. In total, 12 different designs of Ca–alginate capsules were synthesised and identified as X-Y-Z, with X being the type of oil encapsulated (VCO, WCO, or VEO), Y representing the alginate concentration (2% or 3%), and Z referencing the encapsulation method (M1 or M2). For example, capsules synthesised with VEO, 2% alginate concentration, using the syringe pump method is labelled as VEO-2-M2.

The morphology of the capsules was characterised by their size and shape. The size was statistically evaluated as the diameter of 100 randomly selected capsules using an optical microscope (Leica EZ4, Wetzlar, Germany) with 35× magnification and the image processing software ImageJ^®^. The shape of each capsule design was also evaluated by the sphericity factor ($S_{F}$) as specified by Alpízar-Reyes et al. [19], where $S_{F}$ values below 0.05 indicate particles tending to a spherical shape, while values closer to 1 indicate particles with an elongated morphology. The encapsulation efficiency (E.E), i.e., the proportion between the encapsulated oil and the total oil to synthesise the capsules, was determined in this study based on the method described by Concha et al. [25].

The relative density of the capsules was measured following method B of the ASTM D792-13 [33]. Thermogravimetric analysis (TGA) tests were carried out for each design of capsule and their components. For this, a thermogravimetric analyser (NETZSCH, STA 409 PC, Wittelsbacherstraße, Germany) was used. An amount of ~25 mg was placed in a ceramic cap and heated from ambient temperature to 600 °C at a rate of 10 °C/min. All the tests were performed under a constant N_2_ (99.9995%, Airliquide, Santiago, Chile) flow of 50 mL/min, ensuring an inert atmosphere during the test.

The mechanical characterisation of each capsule design was performed by compressive strength and micro-indentation under conditions previously tested by Norambuena-Contreras et al [21]. Compressive tests were performed under ASTM D695-02a [34] using a Universal Testing Machine (Test Resources, Shakopee, MN, USA) equipped with parallel plates and a 1 kN load cell, and operated a at a speed of 0.2 mm/min. The hardness was measured on each capsule design using an HV-1000A Microhardness Tester (Russell Fraser Sales Pty Ltd., Kirrawee, New South Wales, Australia) equipped with a Vickers indenter probe. Representative compression and hardness values were obtained as the average of 10 and 5 measurements, respectively.

### 2.5. Self-Healing Efficiency of the Oils as Bitumen Rejuvenators

The self-healing efficiency of each oil was quantified in cracked bitumen samples by fluorescence microscopy. For each of the oils, a thin film bitumen sample with dimensions 20 × 20 × 0.5 mm^3^ was prepared on a glass petri dish using masking tape and a metallic spatula. A 200 µm-width microcrack was made in the centre of the sample using a blade feeler gauge. Then, using a hollow needle, a drop of ~2 mg of each oil was dropped on the microcrack simulating the release of the rejuvenator from the capsule. The crack closure was recorded periodically every 1 min until its closure using an inverted fluorescence microscope (ICOE IV 5100 FL, Ningbo, China) with phase contrast and a magnification up to 400×. The representative crack width at each time was quantified as the average of 8 measurements along the crack’s width using image processing software ImageJ^®^ Fiji distribution, version 1.52 p (MPI-CBG, Dresden, Germany). Finally, the self-healing efficiency of each oil was calculated as the relationship between the average crack width at a specific time measured in μm, and the initial crack width measured in μm.

### 2.6. Statistical Analysis

The simultaneous effect of the type of oil, the alginate concentration, and the synthesis method on the morphological, physical, and mechanical properties of the capsules was evaluated. A general factorial design 2 × 2 × 3 was proposed for the design of the capsules, consisting of 2 levels of alginate concentrations (2% and 3%), 2 levels associated with the synthesis method (M1-dropping funnel and M2-syringe pump), and 3 levels associated with the type of oil (VCO, WCO, and VEO). Analysis of variance (ANOVA) was used to evaluate the statistically significant effect of the main and combined factors (α = 0.05). Tukey HSD Post-Hoc test was conducted to evaluate pairwise mean comparisons with a significance of α = 0.05. All the statistical analyses were performed using the software OriginPro 2022, version 9.95-2022b (OriginLab Corporation, Northampton, MA, USA).

## 3. Results and Discussion

### 3.1. Effect of the Oil Type and Alginate Concentration on the Physical Properties of the Emulsions

Table 2 shows the average results of the viscosity for the different O/W emulsions. It can be noticed that the viscosity values were significantly affected by the type of oil and the alginate concentration. In particular, the higher the viscosity of the oil (see Table 1) and the alginate concentration, the higher the viscosity of the O/W emulsion was. 

The viscosity of the O/W emulsion is a crucial parameter during the encapsulation process, since it can affect the release frequency of the droplets from each encapsulation device, influencing the morphology of the capsules, e.g., their size and shape. In particular, it is hypothesised that the extrusion of highly viscous emulsions retards the release of the outgoing droplets from each encapsulation device, resulting in bigger and elongated Ca–alginate capsules. This hypothesis is evaluated in Section 3.2.

**Figure 2:**
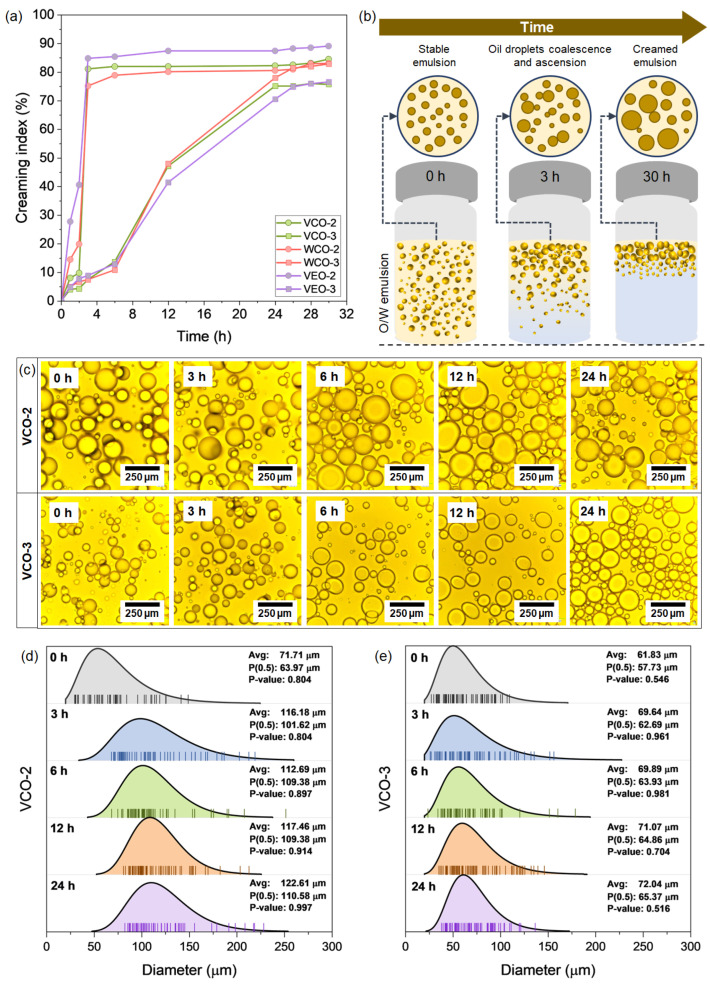
(**a**) Average results of creaming index evaluated at different times for the different O/W emulsions; (**b**) schematical representation of the creaming process occurring in the O/W emulsions over time; (**c**) examples of fluorescence microscopy images for the VCO-2 and VCO-3 emulsions showing the oil droplet growing over time; and statistical distribution of the oil droplet size over time for the case of (**d**) VCO-2 and (**e**) VCO-3 emulsions.

Another important factor to evaluate in the O/W emulsions is their physical stability during the encapsulation process, i.e., the disperse phase (oil) should be homogeneously distributed into the continuous phase (alginate solution). Thus, the physical stability of the emulsions was estimated by analysing the creaming index (CI) values over time (Figure 2a). Results indicate that CI increased with time, suggesting that after a certain period the emulsion components in all the formulations were separated. It was found that emulsions with the same alginate concentration present similar creaming behaviour, suggesting an increase in the stability with the increase of alginate content in the mixture, regardless the nature of the oil.

For O/W emulsions containing 2% of alginate, the increase in the CI with time can be characterised in two stages: A first stage, from 0 to 3 h, where the CI of the VCO-2, WCO-2, and VEO-2 emulsions sharply increased up to values of 81.14%, 75.2%, and 84.85%, respectively. This indicated an early and fast tendency of the O/W emulsions to be creamed. Then, from 3 to 30 h, there was a quasi-stationary stage with CI values of 84.57%, 83.05%, and 89.14% at 30 h for the VCO-2, WCO-2, and VEO-2 based emulsions, respectively. In contrast, emulsions synthesised with 3% of alginate presented a CI increase defined by four stages: A first stage, from 0 to 6 h was identified, where the CI reached values of 13.71%, 10.8%, and 12.85% for the VCO-3, WCO-3, and VEO-3 emulsions, respectively. Then, for the following three stages (6–12 h; 12–28 h; 28–30 h), the CI was increased with time, but at a lower rate at each stage. At the end, the VCO-3, WCO-3, and VEO-3 emulsions presented CI values of 75.71%, 82.85%, and 76.57%, respectively. Based on the previous analysis, the synthesis of O/W emulsions based on 3% alginate were physically more stable when compared with those based on 2% alginate.

The influence of the alginate concentration on emulsion stability can be explained by several factors. Mainly, the creaming effect involves the coalescence and ascension of larger oil droplets (i.e., disperse phase) to the top of the emulsion over time, as represented in Figure 2b. Since alginate acts as a weighting agent on an O/W emulsion, the higher the alginate concentration in the emulsion, the more difficult the coalescence and ascension of the larger oil droplets to the top of the emulsion. This phenomenon is attributed to the increase in the viscosity of the emulsion, reducing the movement of the oil droplets to coalescence. To prove this point, Figure 2c shows fluorescence microscopy images for the VCO-2 and VCO-3 emulsions taken from the top of the emulsion at 0 h, 3 h, 6 h, 12 h, and 24 h. Figure 2d,e shows the statistical distribution of the droplet size. Since CI was more affected by the alginate concentration than by the type of oil, fluorescence microscopy images of VCO with different alginate concentrations well-represents the creaming phenomenon in all the O/W emulsions. From these images, the increase of the oil droplet size over time can be noticed.

Nonetheless, VCO-3 emulsion presented droplet sizes significantly lower than VCO-2. As example, for the VCO-2 emulsion, the oil droplet size increased from 63.97 µm to 110.58 µm after 24 h, representing an increase of ~73% with respect to the initial size. For the same period of time (24 h), the representative droplet size for the VCO-3 emulsion increased from 57.73 µm to 65.37 µm, representing an increase of ~13% with respect to the initial size. Finally, based on the previous analysis, it can be concluded that the component of the O/W emulsions mainly contributing to their physical stability is the alginate concentration. Additionally, for a successful synthesis of the capsules, the encapsulation should take place once the freshly O/W emulsions are prepared, reducing the occurrence of creaming phenomenon.

### 3.2. Morphological and Physical Properties of the Biocapsules

**Figure 3:**
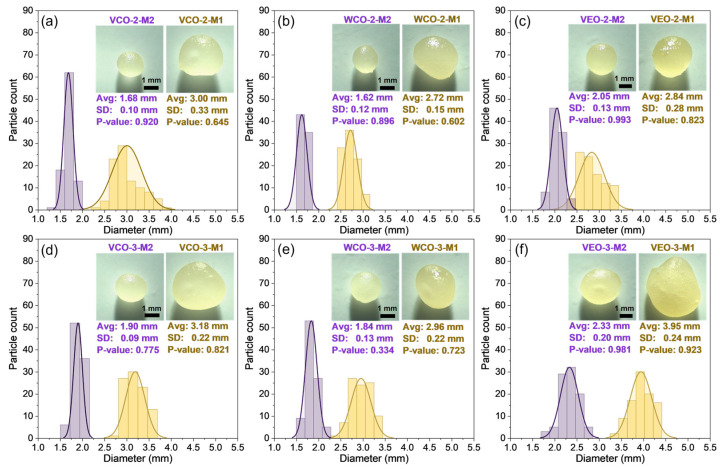
Statistical distribution of the size of each type of capsule fitted to a normal distribution: (**a**) VCO-2-M1 and M2; (**b**) WCO-2-M1 and M2; (**c**) VEO-2-M1 and M2; (**d**) VCO-3-M1 and M2; (**e**) WCO-3-M1 and M2; (**f**) VEO-3-M1 and M2.

Figure 3 shows optical microscopy images and the histograms of the size of each type of capsule fitted to a normal distribution. It can be noticed that the size of the capsules was influenced by the synthesis method, the type of oil, and the concentration of the alginate biopolymer. In particular, (i) capsules synthesised using M1 resulted in bigger sizes than using M2; (ii) capsules with 2% alginate resulted in smaller sizes than those with 3% alginate; and (iii) the VEO capsules presented the biggest sizes followed by the VCO and WCO capsules. As an example, the VEO-3-M1 and the WCO-2-M2 capsules presented the biggest and the smallest sizes with values of 3.95 mm and 1.62 mm, respectively.

**Figure 4:**
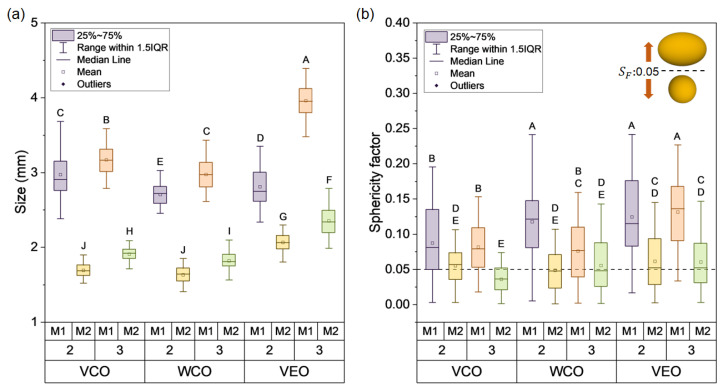
Box plot graphs for (**a**) the size and (**b**) sphericity factor of the different capsule designs.

In particular, Figure 4a represents the size of the capsules through boxplots, where the effect of the encapsulation method on the size of the capsules can be clearly noticed by identifying two groups: capsules synthesised using M1, and capsules synthesised using M2. Moreover, from this Figure we noticed that M1 capsules presented more dispersion on their size when compared to M2 capsules. This can be attributed to the fact that M2 was an extrusion-controlled process, where a constant pressure was applied during the extrusion of O/W emulsions to form the capsule, while for M1 the extrusion mechanism was gravity-based, meaning low control of the release of the O/W emulsion droplets during the extrusion process. Thus, the encapsulation method M2 results in capsules with a more uniform size than M1, and so, a similar shape is expected for M2-type capsules.

To prove the previous hypothesis, Figure 4b shows the average $S_{F}$ results of the capsules. From this Figure, capsules synthesised using M1 tended to $S_{F}$ values over 0.05, indicating a more elongated morphology, while capsules synthesised under M2 presented lower dispersion of data with $S_{F}$ values closer to 0.05, indicating that this encapsulation process resulted in capsules with more regular spherical morphology. The alginate concentration and the type of oil also influenced the morphology of the capsules. Thus, capsules synthesised with highly viscous oils and higher concentrations of alginate resulted in more elongated morphologies. Finally, considering that the capsules are added as an additive to asphalt mixtures, bigger sizes with elongated morphology could affect the physical properties of the asphalt mixture more significantly than smaller sizes and regular spherical morphology. Thus, capsules based on VCO or WCO, 2% alginate and method M2 are recommended for addition in asphalt mixtures.

**Figure 5:**
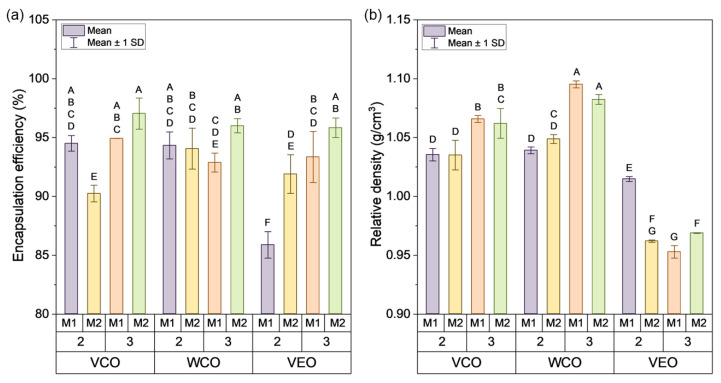
Average results of (**a**) encapsulation efficiency and (**b**) relative density of each capsule.

Otherwise, Figure 5a shows the average results of encapsulation efficiency for each capsule design. It is observed that the capsules presented E.E values ranging from 85.88% (VEO-2-M1) to 97.03% (VCO-3-M2). Overall, the effect of the type of oil on the efficiency can be noticed, observing that VEO capsules presented the lower E.E values when compared to VCO and WCO capsules. This can be attributed to the flow properties, associated with the viscosity of the capsules’ components. As characterised in Section 2.1 and Section 2.3, the viscosity of the VCO and the WCO was lower than the 2% and 3% alginate solutions, respectively. On the contrary, VEO viscosity was higher than the 2% alginate solution, but lower than the 3% alginate solution. This means that for the extrusion process (using M1 or M2) of an O/W emulsion based on 2% alginate, both VCO and WCO will flow more easily than the alginate solution, increasing their E.E values when compared with the VEO. For the extrusion of the O/W emulsion based on 3% alginate, all the oils will flow more easily than the alginate solution, resulting in E.E values higher than for the 2% solution.

In contrast, Figure 5b demonstrates that VEO capsules presented lower density values than the VCO and WCO capsules. Moreover, the increase in the alginate concentration leads to an increment in the density of VCO and WCO capsules, while the VEO capsules exhibited the opposite behaviour being their densities reduced with higher alginate concentrations. These differences in density are ascribed to a combined effect of the sizes and volume of capsules, and to the density of confined oils. According to the data in Figure 4a, the capsule VEO-3-M1 has nearly double the volume (32.3 mm^3^) of capsule WCO-3-M1 (13.7 mm^3^). For the same capsule designs, Figure 5a shows that the VEO-3-M1 and the WCO-3-M1 capsules presented similar E.E values, 93.34% (SD: 2.17%) and 92.87% (SD: 0.8%). Nonetheless, as seen in Table 1, density of VEO is lower than that of WCO. In consequence, the density of VEO-3-M1 capsules is lower than those of WCO-3-M1. From this analysis, it can be noted that the variation in the size of the capsules is higher than that of the E.E. This means that differences in the density of the capsules can be mainly explained by significative changes in their volume.

To evaluate if the differences evidenced in the morphological (size, sphericity) and physical (density and encapsulation efficiency) properties of the capsules are statistically significant, Table 3 resumes the ANOVA p-value results for the main factors (type of oil, alginate concentration, and synthesis method) and their combinations. These results show that significant differences were found on the morphological and physical properties. Particularly, since the type of oil comprises three levels, significant differences detected by ANOVA can be associated with up to three different pairwise means comparisons (VCO-WCO, VCO-VEO, WCO-VEO). Tukey HSD test revealed that the three pairwise means comparisons were significantly different for each of the morphological and physical properties. Therefore, the effect of VCO, WCO, and VEO on the capsule’s size can be treated as three independent groups. In a similar way, each level of the alginate concentration (2%, 3%) and the encapsulation method (M1, M2) can be treated as independent groups.

In terms of the interactions between the main factors, Table 3 revealed the presence of significantly different double and triple interactions, excepting the double interaction between the alginate concentration and the encapsulation method for the measure of sphericity. Graphically, Figure 4a,b and Figure 5a,b present the Tukey HSD analysis for pairwise mean comparison represented by letters. Thus, mean values that do not share a letter can be considered significantly different (*p*-value < 0.05). As an example of the morphological properties of the capsules, Figure 4a shows that the measure of their size was categorised in 10 groups (represented as single letters from A to J), meaning that the size of 10 from the 12 capsule designs can be considered as significantly different. Accordingly, from this Figure, it can be stated that VEO-3-M1 design presented the biggest size, while capsule designs VCO-2-M2 and WCO-2-M2 presented the smaller size values. As an example of the physical properties of the capsules, Figure 5a revealed that their E.E values can be categorised in six groups (A–F). Particularly, VEO-2-M1 is individualised as one single group (F), resulting in the lowest E.E value. 

Based on the previous analysis, it can be concluded that the type of oil, alginate concentration, and encapsulation method, as well as their interactions significantly influenced on the morphology and physical properties of the capsules. In particular, the combined effect of M2 and 2% alginate for each of the oils produced capsules with (i) the smallest and uniform sizes, (ii) regular spherical morphology, and (iii) high encapsulation efficiencies. A similar effect was observed for capsules synthesised using M2 and 3% alginate. Accordingly, M2 could be considered as a potential encapsulation method for the synthesis of homogeneous-sized capsules. To reduce the effect of the capsule addition on the physical properties of the asphalt mixture, capsules based on VCO or WCO, 2% of alginate, and method M2 are recommended for their incorporation in asphalt mixtures. Nonetheless, to select an appropriate alginate concentration and type of oil, thermal–mechanical stability of the capsules and the rejuvenating effect of each oil on cracked bitumen will be analysed within the next sections.

### 3.3. Thermal–Chemical Characterisation of the Capsules and Their Components

**Figure 6:**
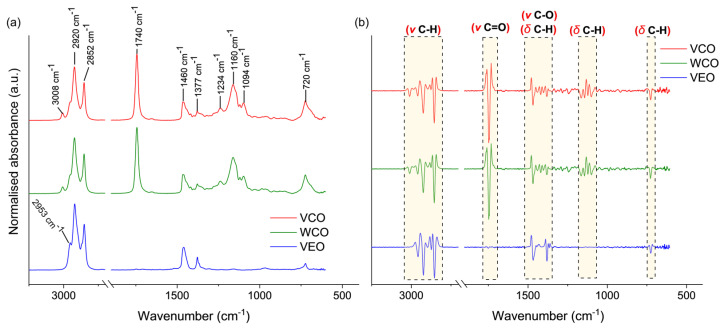
(**a**) FTIR-ATR infrared spectra of VCO, WCO, VEO, and (**b**) second derivative of spectra for VCO, WCO, VEO.

Figure 6 shows the results from the FTIR-ATR characterisation of the rejuvenators used for the encapsulation process. The identification and assignment of spectral bands shown in Figure 6a were represented according to the literature reports [35,36,37] and considering the nature of the rejuvenating liquids. The spectral information gathered from the bioderived oils (i.e., VCO and WCO) were similar, indicating a negligible effect of the oxidation during one cycle of frying for VCO.

Both VCO and WCO presented multiple signals between 1090 cm^−1^ and 1460 cm^−1^, ascribed to C–H bending vibration in aliphatic hydrocarbons, and C–O stretching vibrations commonly found in ethers (see Figure 6b). The signal at 720 cm^−1^ was found in the three oils, and it is typical of weak C–H asymmetric bending in alkyl chains (CH_2_ and CH_3_). In addition, the presence of methylene moieties (CH_2_) in the saturated fatty acid backbone in VCO and WCO was confirmed by the C–H in-plane deformation band at 1377 cm^−1^, and the C–H symmetric and asymmetric stretching bands at 2852 and 2920 cm^−1^, respectively. A distinctive sharp signal was found for VCO and WCO at 1743 cm^−1^. This band is ascribed to the stretching in carbonyl groups (C=O) corresponding to carboxylic acid or triglycerides present in these oils as witnessed before by Goh et al. [37]. In the case of the synthetic VEO, the bands located at 2852 and 2920 cm^−1^ can be associated with the C–H stretching in alkyl chains (CH_2_) [35], while the peaks at 1460 and 1377 cm^−1^ correspond to the C–H bending vibrations in methyl groups typical of synthetic engine oils. As previously stated by Norambuena-Contreras et al. [27], the similarities in intensity, position and nature of functional groups identified in VCO and compared with WCO suggest that WCO is thermally stable, which supports its use as a prospective encapsulated rejuvenating agent in asphalt materials usually manufactured at a temperature >150 °C.

**Figure 7:**
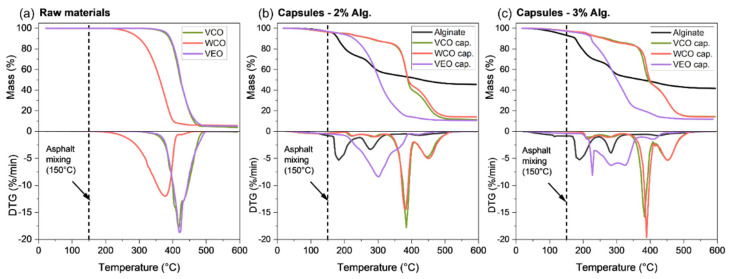
TGA-DTG curves for: (**a**) the VCO, WCO, and VEO; alginate matrix and capsules synthesised with (**b**) 2% and (**c**) 3% of alginate.

Otherwise, Figure 7 shows the TGA-DTG curves for (a) VCO, WCO, VEO, and capsules with, and without, the oils synthesised with (b) 2% and (c) 3% of alginate. As previously recommended by Norambuena-Contreras et al. [30], capsules should be directly added during the asphalt mixing process at a temperature around 150 °C (T_am_). In consequence, it is expected that the capsules as well as their components are thermally stable at this mixing temperature. As seen in Figure 6a, the three oils proposed here as asphalt rejuvenators presented high thermal stability, showing no thermal degradation at T_am_. In fact, these oils started to degrade at temperatures higher than T_am_, with a characteristic single DTG peak identified at temperatures between 200 °C and 450 °C for the WCO and between 320 °C and 500 °C for both VCO and VEO. Although both VCO and WCO come from sunflower, the shifting on the thermal degradation peak of WCO can be attributed to its previous physical–chemical oxidation during cooking. Overall, the single degradation step of VCO and WCO was attributed to the decomposition of volatiles associated to polyunsaturated (linoleic acid) and monounsaturated (oleic acid) fatty acids [38]. From the previous analysis, it is concluded that VCO, WCO, and VEO can be potentially considered as thermally stable rejuvenators for asphalt applications. However, their thermal performance must be proven when encapsulated in the biopolymeric matrix of alginate.

In reference to the encapsulation of the oil in the biopolymeric matrix of alginate, Figure 7b,c show that the TGA curves were quite similar regardless the alginate concentration in the capsules. Regarding the alginate-hollow capsule, its TG behaviour is characterised by two noticeable degradation steps: (i) from 150 °C to 250 °C, related to moisture evaporation and the chemical dehydration of the biopolymer; and (ii) from 260 °C to 350 °C, associated with the fracture of glycosidic bonds, decarboxylation and decarbonylation, releasing H_2_O, CO_2_, and other light compounds [39]. When encapsulating the oils into the alginate biopolymer, Figure 7b,c also show a similar tendency of the capsules with 2% and 3% of alginate when comparing the TGA curves based on the type of oil. Thus, the alginate concentration had no apparent influence on the thermal decomposition of the capsules. Nonetheless, due to the higher thermal stability of the oils, the capsules were significantly degraded at temperatures higher than their respective empty alginate matrices. The particularly earlier thermal degradation for the VEO capsule at ~210 °C can be explained to its low amount of encapsulated oil when compared to VCO and WCO capsules, as demonstrated by the E.E values in Section 2.2. Hence, the enhancement of the thermal stability by effect of the VEO was reduced.

Finally, TGA tests demonstrated that the biopolymeric capsules with VCO, WCO, and VEO can be thermally stable additives for their incorporation to hot mix asphalt (i.e., manufacturing temperatures >150 °C). In particular, capsules based on VCO and 2% alginate could be an optimal design due to: (i) the higher thermal stability of the oil, and (ii) the lower thermal degradation at T_am_. Moreover, since the alginate concentration had no effect on the thermal stability of the capsules, capsule designs with lower alginate concentration could potentially reduce the associated cost of production. Nonetheless, the decision of the appropriate capsule should also consider additional aspects such as the mechanical performance of the capsules and healing capacity of the oil once released from the capsule.

### 3.4. Mechanical Stability of the Biocapsules by Compression and Microindentation Tests

**Figure 8:**
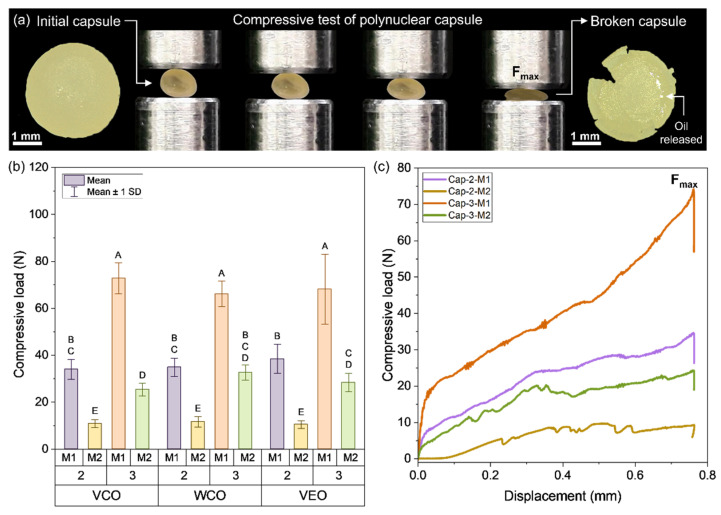
(**a**) Representation of the compressive test carried out on each capsule design; (**b**) average results of the maximum compressive load resisted by each capsule design; (**c**) representative compressive load curves for the capsules depending on the alginate concentration and used method.

In reference to the effect of the main factors studied on the maximum compression load (C_cap_) resisted by each capsule design (Figure 8a,b) shows: (i) a similar tendency of C_cap_ when comparing the capsules based on the type of encapsulated oil, so the compression curves of the capsules can only be represented depending on the alginate concentration and the encapsulation method as shown in Figure 8b. (ii) Moreover, capsules based on 3% of alginate presented C_cap_ values higher than those with 2% of alginate, and (iii) capsules synthesised using M1 presented C_cap_ values higher than those using M2. In consequence, the combination of the factors M1 and 3% of alginate resulted in the highest C_cap_ values. This result can be attributed to the bigger size reached by M1 capsules, distributing the compressive force on a bigger surface than the M2 capsules, and the potential increase of crosslinking reactions between the alginate and the Ca^2+^ ions, producing a strengthening effect on the multicavity structure of the capsule. Based on the previous analysis and supported by Norambuena-Contreras et al. [21], it can be concluded that C_cap_ mainly depended on the synthesis method and the concentration of alginate used to produce the capsules.

Additionally, since the alginate-based capsules are added during the mixing process of asphalt, they should be strong enough to resist the compaction load of the asphalt mixture referenced in 10 N as stated by Ruiz-Riancho et al. in previous research [23]. Figure 8a shows that all the capsules presented values over this limit indicating their suitability to resist the compaction process and to be later activated during the operation of the road by effect of traffic loads. Nonetheless, it should be considered that C_cap_ values far from the previous compaction load reported could difficult the opportune activation of the capsules, reducing the release of the oil on the cracked-aged bitumen. Accordingly, capsules based on M2 and 2% or 3% of alginate could be more appropriate for resisting the compaction process, while being susceptible to the activation process.

**Figure 9:**
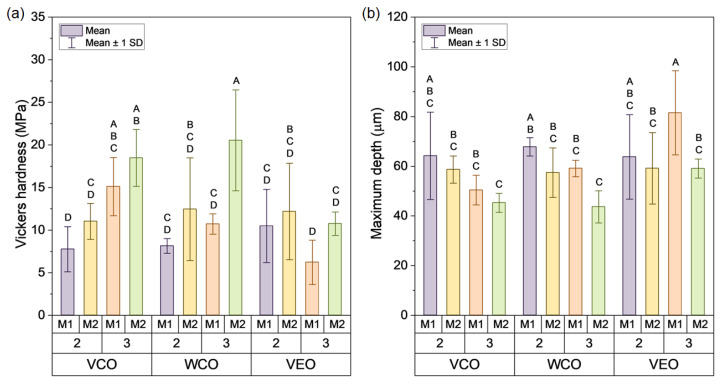
Average results of (**a**) Vickers hardness and (**b**) associated maximum indentation depth.

Similarly, Figure 9a indicates that, like in the compressive tests, the synthesis method and the alginate concentration mainly affected the hardness of the capsules, but in a different way. Particularly, capsules synthesised using method M2 presented hardness values higher than those synthesised using M1. Moreover, the addition of 3% of alginate resulted in higher values of hardness than the capsules with 2% of alginate, also reported in [21]. Thus, the combined effect of M2 and 3% alginate resulted in capsules with the highest Vickers hardness, with an average value of 16.58 MPa (SD: 5.1 MPa) while capsule designs based on M2, and 2% alginate presented an average value of 11.88 MPa (SD: 0.75 MPa). As reference, synthetic polymers used for encapsulation applications present Vickers hardness value around 400 MPa.

In contrast, biopolymers used for encapsulations purposes such as polylactic acid, chitin, and chitosan have been stated in 30 MPa, 57 MPa, and 45 MPa, respectively [40]. Therefore, the use of encapsulating soft materials with lower hardness values, such as alginate, could facilitate oil release from the capsule through its breakage or deformation. This behaviour is precisely what is required in a polynuclear capsule with asphalt self-healing purposes, i.e., that is mechanically resistant to the manufacturing processes of asphalt mixtures, but with the ability to be susceptible to the mechanical activation by the effect of the stress concentration trigger on the surface of the capsule. 

The overall hardness values of the capsules are in line with their indentation depth as shown in Figure 9b, i.e., the higher the hardness, the lower the indentation depth, so the capsule offered more resistance to be penetrated by the indenter. As an example, the WCO-3-M2 capsule presented the highest average hardness and the lowest indentation depth, being 20.53 MPa (SD: 5.92 MPa) and 43.63 µm (SD: 6.45 µm), respectively. In contrast, the VEO-3-M1 capsule presented the lowest hardness and the highest indentation depth, with values of 6.21 MPa (SD: 2.6 MPa) and 81.50 µm (SD: 6.45 µm), respectively.

Differences in hardness values can be partially attributed to the relative densities of each capsule and to a consequence of their microstructures, see Figure 5b. Thus, the denser the capsule, the lower the indentation depth and so the higher the hardness of the capsule. With this antecedent, it is hypothesised that denser capsules based on 3% of alginate mechanically reinforced their biopolymeric microstructure than those with 2% of alginate, being able to better resist the indentation load. Thus, in relation to the raw materials constituting the capsules, it can be concluded that the alginate was mainly responsible of the compressive strength and hardness of the capsules. Thus, based on the previous recommendation on the use of capsules based on M2 and 2% or 3% of alginate, it can be concluded that capsules synthesised using M2, and 2% of alginate are recommended to activate a capsule for self-healing of crack-aged asphalt mixtures.

Table 4 resumes the ANOVA p-value results for the main factors affecting the mechanical performance of the capsules (type of oil, alginate concentration, and synthesis method) and their combinations. Results indicate that the type of oil had no significant effect on the compression resistance of the capsules, while for the Vickers hardness and the indentation depth, the type of oil led to significant differences. For this test, the Tukey HSD analysis revealed significant differences in the VCO-VEO and WCO-VEO pairwise mean comparisons. For each level of the alginate concentration (2%, 3%) and the encapsulation method (M1, M2) the three mechanical variables presented significant differences, excepting the effect of the alginate concentration for the Vickers depth (*p*-value > 0.05). 

Additionally, Table 4 shows that the detection of the double and triple interactions between variables was different for the mechanical variables evaluated. In reference to the compressive load, Table 4 revealed that only the interaction between the type of oil and the alginate concentration had no significant effect (A * B interaction). In the case of the Vickers hardness and Vickers depth, significant differences were only detected for the interaction between the type of oil and the alginate concentration (A * B interaction). Figure 7a and Figure 8a,b present the Tukey HSD analysis for pairwise mean comparison represented by letters. Based on the recommendation on the use of capsules based on 2% or 3% alginate and the synthesis method M2, Tukey HSD analysis revealed that the pairwise mean comparison of 2-M2 and 3-M2 double interactions was significantly different for compression and hardness. This result confirms the selection of the capsule design based on 2% alginate and the synthesis method M2 to facilitate the activation of the capsule.

### 3.5. Healing Capacities of Types of Oils as Asphalt Rejuvenators

**Figure 10:**
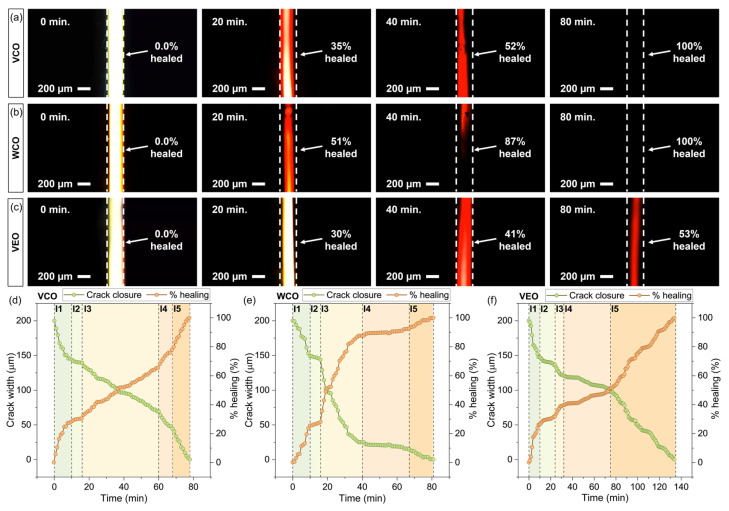
Fluorescence microscopy images for (**a**) VCO, (**b**) WCO, and (**c**) VEO asphalt rejuvenators on asphalt film samples at 0 min, 20 min, 40 min, and 80 min. Results of crack width and healing efficiency of bitumen samples rejuvenated with (**d**) VCO, (**e**) WCO, and (**f**) VEO over time.

Figure 10a–c shows the microscopy fluorescence images of the microcrack closure in bitumen samples by the effect of the rejuvenating oils at 0 min, 20 min, 40 min, and 80 min. From these images, it can be seen that the crack closure was affected by the type of oil used as asphalt rejuvenator, since the crack width over time was not uniform. For a time of 80 min, the µ-crack was completely closed when VCO was used as the rejuvenator. At this time, the bitumen samples with WCO and VEO presented µ-cracks width values of 2.57 µm and 92.62 µm, respectively, suggesting that oil viscosities play a role in this process (Table 1). This behaviour suggests that the more viscous the oil, the lower its diffusion capacity into the cracked zone, resulting in a retardation of its softening effect on the aged bitumen. 

To prove the previous hypothesis, Figure 10d–f show the crack width and the healing efficiency curves of bitumen samples with (d) VCO, (e) WCO, and (f) VEO recorded every 1 min until the complete closure of the crack was attained. Overall, shorter times required to close the µ-crack mean a better healing performance of the oil. The complete crack closure of the bitumen samples by effect of VCO and WCO took 78 and 81 min, respectively. Those times were considerably lower than that of the VEO, requiring a time of 135 min for complete crack closure. Based on this analysis, VCO could be more appropriate as an encapsulated rejuvenator for asphalt self-healing purposes. 

Nonetheless, Figure 10d–f also show that the µ-crack closure over time was not uniform for the different oils used. The variability of the µ-crack closure over time can be grouped by five different intervals characterised as a linear relationship between the crack width and time. Since each of these intervals took place in different periods of time, a rate of crack closure ($\left|R_{c}\right|$) was calculated for comparison purposes, as depicted in Equation (1):

(1) $$\left|Rate\;of\;crack-closure\;\left(R_{c}\right)\right|=\frac{cw_{f}-cw_{i}}{tw_{f}-tw_{i}}$$

where *cw_i_* and *cw_f_* are the initial and final crack width values for each interval, in µm, respectively, and *tw_i_* and *tw_f_* are the associated initial and final times, in min.

Table 5 summarises the $\left|R_{c}\right|\;$ values for each bitumen sample. Overall, $\left|R_{c}\right|$ values allow to identify (i) changes in the healing performance of each oil, quantifying them and (ii) the time periods where each of the oils are more efficient to heal the cracked bitumen (e.g., at the beginning or the end of the healing process). In consequence, the higher the $\left|R_{c}\right|$ value, the faster the difussion of the oil and so the more effective the healing process. From Table 5, it was noticed that all the samples presented quite similar crack closure rates during the first 10 min. This means that the oils, during the first interval, presented an initial softening effect on the bitumen in direct contact with them. In this initial stage, the viscosity of the oils was not a determinant factor, since all the oils reduced the initial crack from 200 µm to 150 µm in a period of 10 min (crack closure rate around −5 µm/min). 

During the second interval, the crack closure rate was significantly reduced to a quasi-stationary state. This meant that the oils were diffused into the bitumen surrounding the microcrack to close it. Thus, from the second interval, the viscosity of each oil is a determinant factor for the crack’s closure over time. For instance, Table 5 shows that, for each oil, the $\left|R_{c}\right|$ values were quite similar with values varying between 0.78 µm/min and 0.87 µm/min. Nonetheless, this quasi-stationary condition was maintained for B–VCO and B–WCO for 6 min, while during 14 min for the B–VEO. Hence, the higher the viscosity of the oil, the longer the duration of the quasi-stationary state.

From the second interval to the final one, the B–VCO sample increased its crack-closure rate from 0.87 µm/min to a maximum value of 4.42 µm/min following a linear tendency, while for B–WCO and B–VEO samples, the crack closure rate was quite irregular with alternating values. This indicates a more consistent softening effect of VCO, constantly diffusing into the bitumen surrounding the µ-crack over time. Based on these results, it can be concluded that VCO and WCO showed better healing performance than VEO. In particular, the shorter time taken by VCO to close the initial 200 µ-crack and its better performance during the crack-healing process indicate its potential use as an effective rejuvenator to be encapsulated for asphalt self-healing purposes.

## 4. Conclusions

In this paper, a factorial approach was used to evaluate the effect of the capsules’ design parameters on the physical, thermal, and mechanical properties of enhanced encapsulated rejuvenators for asphalt self-healing. The main findings revealed that using an extrusion-controlled method, such as a syringe pump, was the best for producing homogeneously sized capsules. Capsules synthesised with lower alginate concentrations and VCO as rejuvenator meet appropriate thermal and mechanical stability criteria for their incorporation into bituminous materials. The design parameters and their interactions also affected the properties of the biobased capsules and their components, concluding that:

- Variations in the physical stability of an O/W emulsion were mainly explained by changes in the alginate concentration reduced the creaming rate with time. Creaming was not affected when varying the type of oil.
- It was concluded that to avoid any potential phase separation of the emulsion components, it is recommended that the encapsulation process take place once prepared the O/W emulsions.
- The use of the syringe pump method and low alginate concentrations produced capsules with the smallest and uniform sizes (1.60–1.68 mm), regular spherical morphology and high encapsulation efficiencies, up to 94%.
- Encapsulation efficiency of the capsules was affected by the viscosity of the capsule’s components. Overall, high encapsulation efficiencies were reached when the viscosity of the oil was lower than the alginate solution (e.g., VCO-based capsules).
- Thermal stability of the capsules mainly depended on the type of oil used as rejuvenator. Capsules based on VCO presented the best thermal stability with mass losses below 5% at the temperature of asphalt mixing (150 °C) as seen by TGA test.
- FTIR-ATR results showed that the spectral information gathered from the bioderived oils (VCO and WCO) was similar, indicating a negligible effect of the oxidation during one cycle of frying for VCO.
- Capsule design based on the syringe pump method (M2) and 2% alginate showed compressive strength and hardness values of 10 N and 12 MPa, respectively, potentially surviving the compaction process of bituminous materials and its later activation under traffic loads.
- Healing tests on cracked bitumen samples revealed the use of VCO reduced the time to close a 200 microcrack with better performance during the crack-healing process, indicating its potential use as an effective rejuvenator for asphalt self-healing.
- Finally, the synthesis of capsules under the syringe pump method, using virgin cooking oil (VCO) and 2% alginate, as an effective encapsulated additive to improve the self-healing properties of bituminous materials is recommended.

Finally, the interactions of the capsules’ design parameters discovered in this study open new research on the use of factorial designs to synthesise more capsules for asphalt self-healing focused on: (i) the optimisation of the design parameters of the capsules such as the concentration of CaCl_2_ and the concentration of the encapsulated oil, (ii) the study on the proper amount of oil to rejuvenate long-term aged bitumen based on rheological tests and its equivalent dosage in capsules, and (iii) the effect of the content of optimised capsules on the physical, mechanical, and healing properties of bituminous materials.

## Figures and Tables

**Table 1:** Physical properties of the oil used as bitumen rejuvenators.

Type of Oil	Density (g/cm^3^)	Viscosity at 20 °C (cP)	pH at 25 °C
VCO	0.85	70	5.3–5.5
WCO	0.83	89	4.4–4.6
VEO	0.73	274	7.9–8.1

**Table 2:** Average results of viscosity values of the O/W emulsions measured @20 °C, in cP.

VCO-2	VCO-3	WCO-2	WCO-3	VEO-2	VEO-3
245.5 ± 7.2	824.1 ± 9.2	270.0 ± 9.3	940.0 ± 7.7	291.1 ± 11.3	1053.0 ± 8.4

**Table 3:** ANOVA p-values showing the effect of the main studied factors and their interactions on the morphological and physical properties of the capsules.

Variables	Size	Sphericity	Density	Encapsulation Efficiency
A	<0.001 ^(1)^	<0.001 ^(1)^	<0.001 ^(1)^	<0.001 ^(1)^
B	<0.001	<0.001	<0.001	<0.001
C	<0.001	<0.001	0.0012	<0.001
A * B	<0.001	0.0028	<0.001	<0.001
A * C	<0.001	<0.001	0.0029	<0.001
B * C	<0.001	0.0725	0.0016	0.0160
A * B * C	<0.001	<0.001	<0.001	<0.001

Notes: A: oil type (VCO, WCO, VEO); B: alginate concentration (2%, 3%); C: encapsulation method (M1, M2). ^(1)^: significant differences for VCO-WCO, VCO-VEO, and WCO-VEO by Tukey HSD mean comparisons.

**Table 4:** ANOVA p-values showing the effect of the main studied factors and their interactions on the mechanical properties of the capsules.

Variables	Compressive Load	Vickers Hardness	Vickers Depth
A	0.8838	0.0141	0.0039
B	<0.001	0.0013	0.0575
C	<0.001	<0.001	<0.001
A * B	0.5128	<0.001	0.0026
A * C	0.0238	0.1800	0.4046
B * C	<0.001	0.1501	0.1767
A * B * C	0.0159	0.5225	0.3859

Notes: A: oil type (VCO, WCO, VEO); B: alginate concentration (2%, 3%); C: encapsulation method (M1, M2).

**Table 5:** Results of $\left|R_{c}\right|$ values in µm/min for each interval and rejuvenated bitumen.

Sample	Interval 1	Interval 2	Interval 3	Interval 4	Interval 5
Bitumen–VCO	5.62	0.78	1.59	3.12	4.42
Bitumen–WCO	5.09	0.87	4.96	0.37	1.06
Bitumen–VEO	5.25	0.79	1.95	0.54	1.63

## Data Availability

Not applicable.
